# Microbiological evaluation of conjunctival anopthalmic flora after using digital 3D-printed ocular prosthesis compared to conventional one: a randomized clinical trial

**DOI:** 10.1186/s12903-023-03746-w

**Published:** 2023-12-18

**Authors:** Yassmin A. Tahmawy, Faten S. Mohamed, Suzan Elfeki, Mervat E. Abd-ELLAH

**Affiliations:** 1 Department of Prosthodontics, Faculty of Dentistry, Alexandria University, Champollion Street, Azarita, Alexandria, 002034868066 Egypt; 2 Department of Microbiology, Faculty of Medicine, Alexandria University, Alexandria, Egypt

**Keywords:** Ocular prosthesis, 3D printing, Anophthalmic flora, Computer aided

## Abstract

**Background:**

This study aims to assess the influence of using 3D-printed acrylic resin versus conventional Poly-methyl methacrylate (PMMA) for fabricating ocular prostheses on the biofilm and microbial flora of anophthalmic socket.

**Methods:**

A randomized controlled trial was designed as a parallel group study. Participants were allocated randomly into two groups: the control group, which received conventionally fabricated ocular prostheses (CG, *n* = 11), and the test group, which received digitally 3D-printed ocular prostheses (DG, *n* = 11). Microbiological analysis was conducted before prosthesis insertion and three months after using the ocular prosthesis. Swab samples were inoculated on blood agar, MacConkey’s agar, and Sabouraud’s dextrose agar (SDA) for isolating Gram-positive, Gram-negative, and fungal organisms, respectively. Subsequently, the plates were incubated at 37 degrees Celsius for 48 h. Additionally, a validated questionnaire was used for subjective clinical evaluation, including parameters such as comfort level, socket discharge, lacrimation, and frequency of lubrication for each ocular prosthesis patient in both groups.

**Results:**

Test group (DG, *n* = 11) exhibited a positive, though statistically insignificant, difference (*p* > 0.001) in microbial growth when compared to the control group (CG, *n* = 11). A statistically significant difference was observed in comfort levels between the two groups, with more comfort level within group II (test group) patients. While parameters such as discharge amount, discharge location, lacrimation and lubrication frequency displayed statistically insignificant differences between the two groups, all parameters showed improved results after three months of prosthesis use.

**Conclusions:**

The choice of ocular prosthesis fabrication technique did not yield a statistically significant difference in anophthalmic flora. However, the 3D-printed acrylic resin, as an artificial eye material, displayed potential advantages in reducing the colonization of opportunistic pathogens. All subjective clinical evaluation parameters exhibited enhanced outcomes after three months of prosthesis use, emphasizing the need for an adaptation period during which patients complains are alleviated. In comparison with PMMA, 3D-printed acrylic resin showcased a certain degree of anti-colonization ability against pathogenic bacteria, along with a significant level of patient comfort, suggesting its potential as a promising material for ocular prostheses.

**Trial registration:**

This parallel double-blinded RCT has been registered at ClinicalTrials.gov with identification number: NCT05584865, 18/10/2022.

## Background

The objectives of treating anophthalmic patients with ocular prostheses are to restore facial aesthetics and self-esteem. This involves repairing eyeball deformities, protecting the anophthalmic cavity, preserving the palpebral muscle tone, inhibiting palpebral collapse, restoring lachrymal direction, and preventing the accumulation of lachrymal fluid within the eye cavity [1].

However, wearers of ocular prostheses encounter varying degrees of comfort. Often, they exhibit symptoms of chronic discharge and irritation. Several factors have been proposed as underlying causes of these issues. One such factor is the alterations that occur in the conjunctival flora after enucleation and subsequent use of ocular prostheses [2].

Numerous studies have investigated the microbial composition of the conjunctival flora within the anophthalmic cavity. The majority of these studies have demonstrated higher rates of pathogens in the socket compared to the normal conjunctival flora [3–6].

These conspicuous changes in the socket flora may arise due to modifications in the conjunctival epithelium following enucleation or evisceration surgery. The absence of the globe and the use of an artificial eye led to various alterations on the ocular surface. Firstly, the bulbar conjunctiva is no longer swept by the eyelids. Secondly, the ocular prosthesis, functioning as a foreign body, can cause frictional irritation of the conjunctiva during its movement [7]. Additionally, the extensive manipulation of the cavity while placing and removing the prosthesis transports external microorganisms into the conjunctival sac, increasing the likelihood infections developing [8, 9].

Furthermore, the presence of dead space [10] between the posterior surface of the prosthesis and the anterior surface of the socket allows for the accumulation of conjunctival debris and tear secretion. This space, along with mucous, provides an ideal environment for bacterial growth. The existence of dead space is primarily related to ill-fitting prostheses, which heavily depend on the method of prosthesis construction. The conventional ocular prosthesis is typically created from an impression of the anophthalmic cavity, which can encounter challenges such as displacement of the impression material by surrounding tissues, sensitivity to impression materials, excessive pressure on anophthalmic tissues, and occasionally an inability to reach the socket’s boundaries [11].

A significant advancement in the realm of customized ocular prostheses is the utilization of digital technology, including Computer-Aided Designing (CAD) and Computer-Aided Manufacturing (CAM), which hold the key to creating accurate and aesthetically pleasing ocular prosthesis. Few studies have explored the construction of digital ocular prostheses, all of which have shown promising results in terms of aesthetics, fit, and patient satisfaction [7–9].

Finally, some studies suggest that the material and surface roughness of ocular prosthesis can influence interactions with microorganisms, potentially leading to the establishment of biofilm bacteria, which may, in turn, result in infections. As for the material used in the fabrication of ocular prostheses, thermally activated acrylic resin remains the most commonly employed material in clinical practice [12].

Acrylic resin is the most commonly used material for fabricating artificial eyes due to its excellent physical and mechanical properties [13–15]. It is also biocompatible with the tissues surrounding the ocular conjunctiva, providing an aesthetically pleasing, scratch-resistant, and well-polished prosthesis. However, it does have some disadvantages that require attention, including dimensional inaccuracy and polymerization shrinkage.

Poly-methyl methacrylate (PMMA) is another material used, but it has its drawbacks. It is a porous material that is prone to microbial biofilm accumulation [16]. Additionally, the poor wettability and hydrophobicity of PMMA surfaces can contribute to various anophthalmic socket disorders, such as dryness, lacrimal drainage blockage, excessive mucoid discharge [17].

With the introduction of computer-aided design and computer-aided manufacturing (CAD/CAM) technology in the fabrication of ocular prostheses, 3D-printed acrylic resin has become a notable material choice. Generally, 3D-printed resins show promise for clinical applications due to their improved mechanical and physical properties, making them suitable for replacing conventional fabrication techniques [18, 19]. Printable resins composed of photosensitive (also optional thermosetting) liquid monomers. Printed elements are post-cured in an ultraviolet (UV) oven to obtain additional cross-linking of the unreacted monomer chemical groups which improve their mechanical properties and also increase resins biocompatibility.

Several studies have compared the different properties of 3D-printed photopolymerized resin to conventional PMMA resins in applications such as denture bases, temporary crowns, and orthodontic appliances [19, 20]. However, direct comparisons of the biological behavior between conventional acrylic and 3D-printed resin as materials for ocular prostheses are rarely conducted.

Therefore, this study aims to evaluate the microbiological properties of digitally designed 3D-printed biocompatible resins in comparison to conventionally manufactured heat-cured acrylic resin, which is the most commonly used material for ocular prostheses. The objective is to gain a better understanding of the pathophysiology of infections in the anophthalmic cavity when using different types of ocular prostheses designs and materials. The null hypothesis of this study posits that there is no difference between the microbiotas of anophthalmic socket after use of both types of ocular prostheses material.

## Methods

### Study setting

This clinical and microbiological comparative study took place at the Department of Prosthodontics, Faculty of Dentistry, and the Department of Microbiology, Medical Research Institute, Alexandria University. This trial has been registered at ClinicalTrials.gov with identification number: NCT05584865, registration date: (18/10/2022). The authors certify that this trial has received ethical approval from the Research Ethics Committee, Faculty of Dentistry, Alexandria University, Egypt (international No.: IORG0008839, ethics committee number: 0441–6/2022; date of registration: 9/6/2022). Signed written informed consent forms were obtained before enrollment in this trial.

### Sample size calculation

The sample size was calculated with 5% alpha error and 80% study power. Hashem et al. [21] reported an excellent comfort level in 10 patients (83.3%) who received the digital ocular prosthesis compared to only one patient (8.3%) of those who received the conventional ocular prosthesis. Based on a comparison of two independent proportions, the minimum sample size was calculated to be 10 patients per group, increased to 11 to account for potential loss to follow-up. Therefore, the total required sample size = number of groups x number per group = 11 × 2 = 22 patients. The sample size was calculated by using G*Power 3.1.9.7 [22].

### Inclusion and exclusion criteria

All subjects enrolled in the study had unilateral anophthalmic socket. The subject-related inclusion criteria were: (i) age range between 19 and 65 years; (ii) Had enough ocular bed to accommodate the ocular prosthesis; (iii) At least 3 months after surgery to allow complete healing of the socket. Subjects were excluded if they were: (i) Under steroid therapy or those under radio or chemotherapy; (ii) had eye lid deformity; (iii) Had history of psychological disorder or systemic disease.; (iv) had eye infection.

### Study design

**Fig. 1:**
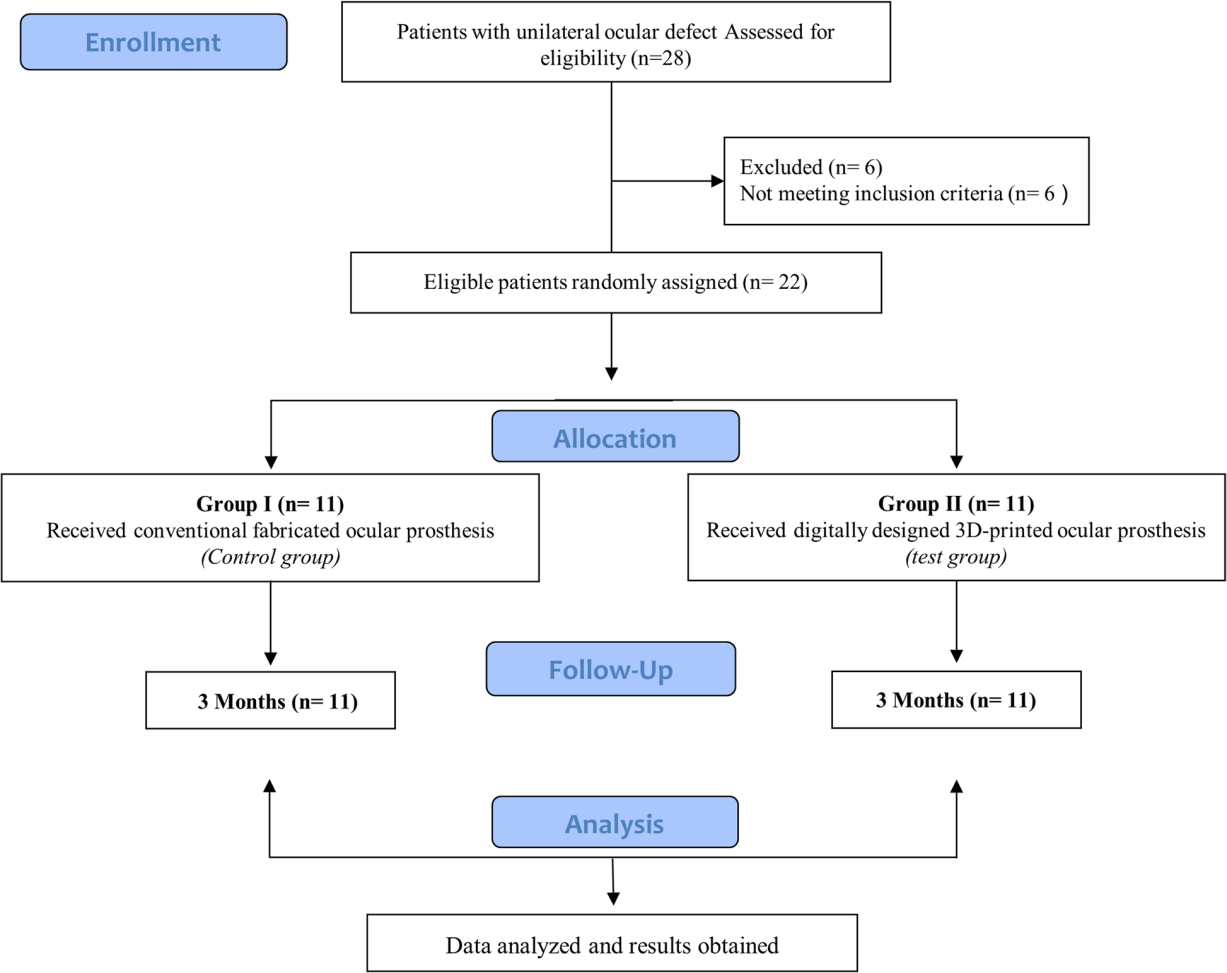
Flow diagram of study design following CONSORT guidelines

This is a randomized, parallel control trial with two balanced parallel arms, following the CONSORT checklist [23]. A random allocation sequence was generated using an online software program (Research Randomizer; http://www.randomizer.org) [24]. Randomization sequence in blocks of 2 was created using randomization software (Sealed Envelope, London, UK). The allocated group was written down on a piece of paper that was enfolded in an opaque sealed envelope with the patient’s respective number at time of evaluation. The treatment allocation was performed by an assistant who was not part of the study. Twenty –eight Patients with unilateral ocular defects were selected to participate in this study from the Maxillofacial Clinic, Department of Prosthodontics, Faculty of Dentistry, Alexandria University, from November 2022 to April 2023. Twenty-two participants were eligible to enter the study based on the inclusion and exclusion criteria (Fig. 1). That study aimed to investigate the relationship between conjunctival flora of anophthalmic sockets and the design and material of ocular prostheses. All patients received their prostheses for the first time, all patients followed the same cleaning method for their ocular prostheses, using warm water and plain soap, and all had their prosthesis removed and cleaned once a week. No ocular prostheses in either group were polished from the time of insertion until the final swab was taken after 3 months of insertion and none were using any type of ophthalmic medication.

After obtaining informed consent and a patient’s medical history, epidemiological data of interest were collected including age, gender, eye loss, etiology of eye loss, and occupation as presented in (Table 1).

**Table 1:** Patient demographic data for group I and II

**Group I (Conventional Group)**										
**Patients Number**										
**1**	**2**	**3**	**4**	**5**	**6**	**7**	**8**	**9**	**10**	**11**
**Age (Years)**										
18	19	55	62	63	65	42	21	32	35	40
**Gender**										
Male	Male	Female	Female	Female	Male	Male	Female	Male	Male	Male
**Lost Eye**										
Right	Left	Left	Left	Right	Right	Left	Right	Right	Left	Right
**Time Since Eye Loss (Months)**										
6	8	12	6	6	3	3	4	6	3	6
**Cause of Eye Loss**										
Trauma	Trauma	Failed surgery	Infection	Infection	Failed surgery	Trauma	Trauma	Trauma	Trauma	Trauma
**Occupation**										
Student	Student	Farmer	Farmer	Housewife	Retired	Laborer	Housewife	Laborer	Technical	Technical
**Group II (Digital Group)**										
**Patients Number**										
**1**	**2**	**3**	**4**	**5**	**6**	**7**	**8**	**9**	**10**	**11**
**Age (Years)**										
30	93	60	55	37	25	19	30	60	55	21
**Gender**										
Male	Male	Male	Male	Male	Male	Female	Female	Male	Female	Male
**Lost Eye**										
Right	Right	Right	Left	Right	Right	Left	Left		Right	Left
**Time Since Eye Loss (Months)**										
4	6	6	3	6	6	3	3	6	6	4
**Cause of Eye Loss**										
Trauma	Trauma	Failed surgery	Infection	Trauma	Trauma	Trauma	Failed surgery	Infection	Failed surgery	Trauma
**Occupation**										
Technical	Technical	Retired	Laborer	Farmer	Laborer	Student	Technical	Retired	Housewife	Farmer

Participants were randomly assigned to two equal groups. Group I, the control group (CG, *n* = 11) included 11 patients who received conventional ocular prostheses constructed using the original conventional technique with heat-cured acrylic resin as the ocular prosthetic material. Group II, the test group (DG, *n* = 11) included 11 patients who received 3D-printed ocular prostheses printed using medical-grade 3D-printed acrylic resin. Prophylactic antibiotic eye drops (TOBRADEXTM Eye Drops. NOVARTIS, Basel, Switzerland) were prescribed for all patients in both groups to be used for just one week after insertion to prevent infection development after prosthesis insertion. Each group was clinically evaluated using a questionnaire one week after prosthesis insertion and again three months later. Microbiological evaluations were performed before prosthesis insertion (pre-prosthetic swab) and after three months of prosthesis use (post-prosthetic swab).

### Ocular Prosthesis Fabrication in Group I (Conventional Method)

In the case of Group I, a conventional custom-made ocular prosthesis was created following the original technique described by Cain [15]. An accurate impression of the patient’s eye socket was obtained using a suitable ocular plastic tray to prepare an appropriate wax model. This wax model was then flasked and processed into heat-cured acrylic resin (Acrostone Dental Factory under exclusive license VIV England). The acrylic resin was chosen to match the color of the sclera of the patient’s healthy eye. The position of the iris and the outer curvature were also determined and placed in the socket for confirmation.

**Fig. 2:**
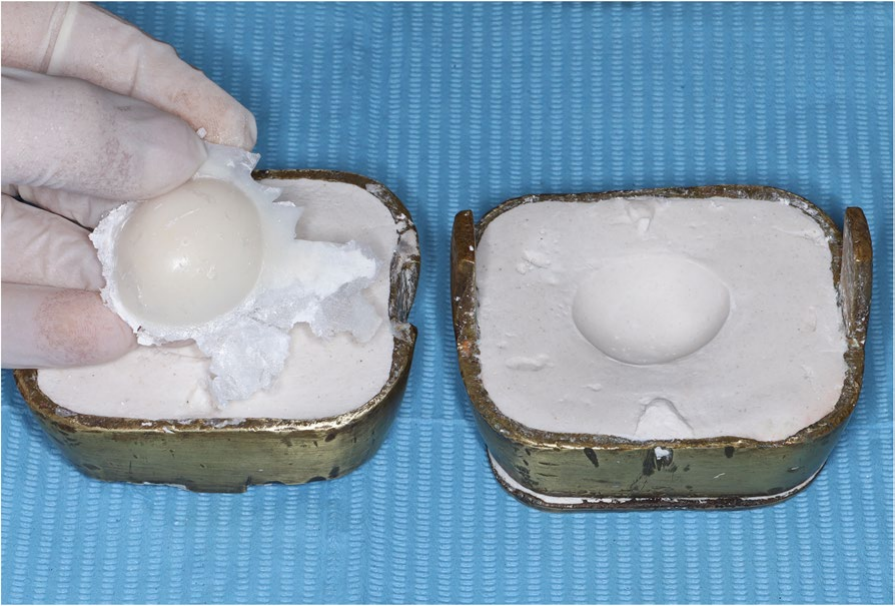
Flasked ocular prosthesis fabricated from PMMA

For the iris disk (Factor II Inc., Lakeside. AZ, USA) was utilized and painted using oil pigments (Gouache oil, Northampton, USA) or earth pigments dissolved in a solvent such as monopoly to achieve the same color as the iris of patient’s healthy eye. A 1 mm reduction was made from the scleral surface to create space for a layer of clear acrylic resin. Subsequently, the sclera was painted and characterized to mimic the appearance of the sclera of the contralateral eye. The iris disk was positioned precisely in the previously determined iris position, and a layer of clear, heat-cured acrylic resin was applied to encapsulate the assembly. The ocular prosthesis was then meticulously finished, polished and subsequently delivered to the patient (Fig. 2).

### Ocular Prosthesis Fabrication in Group II (Digital 3D-Printed Ocular Prosthesis)

In group II, a digitally designed 3D-printed ocular prosthesis was created. A Computerized Tomography (CT) scan image for the orbit was obtained, providing detailed information about the anophthalmic socket in a layered format. Digital Imaging and Communications in medicine (DICOM) data from the CT scan were imported into Materialise’s Interactive Medical Image Control System (MIMICS) software for the segmentation process. This process separates the soft tissue bed of the socket from the eyelids of the affected eye, revealing the fitting surface of the socket. This method replaces the conventional impression technique.

The data was then imported into a 3-Matic software (Materialise 3-matic. CAD link module. Montreal, QC) to create a mid-sagittal plane. A mirrored image of the normal eyeball was used as a reference for the design and construction of the ocular prosthesis for the affected eye. Subtraction the mirrored normal eyeball from the defective eyeball was performed, resulting in the digitally designed ocular prosthesis.

The STL was printed using medical-grade 3D-printed acrylic resin (Preform; Formlabs, USA) on a Formlabs, Form 2 printer (Formlabs, Somerville, USA). This printer operates based on SLA technology with a 405 nm laser wavelength and a layer thickness 50 μm. To ensure that finished parts are biocompatible we followed wash setting and post cure setting as noted in the Instructions for Use, prosthesis washed in 99% isopropyl alcohol for 10 min, then post cured for 60–90 min in 80 °C.

**Fig. 3:**
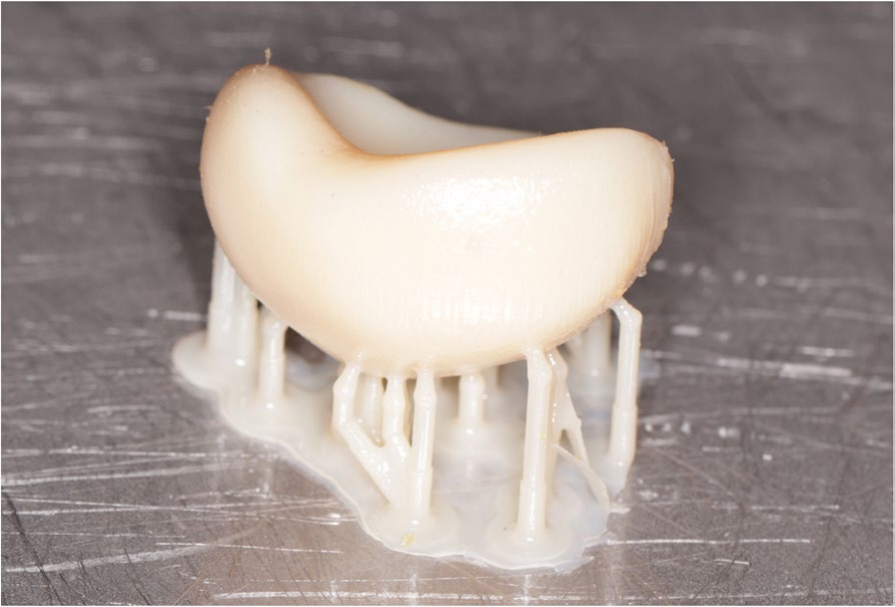
3D-printed ocular prosthesis using medical grade 3D-printed acrylic resin (Preform; Formlabs, USA)

The iris was printed using high-quality patient photographs through UV printing technology, utilizing a Roland Versa UV LEF-200 printer (Roland DG Corp., Japan). Subsequently, conventional sclera characterization was done performed, and layer of clear PMMA was added to cover the sclera, the ocular prosthesis was then finished, polished and delivered to the patient (Fig. 3).

### Subjective clinical evaluation (Questionnaire)

A validated questionnaire was employed to assess the comfort level of the patients. Prior to its use, the questionnaire underwent pilot testing with six prosthodontics experts who were involved in the study. These experts assessed both the content and face validity of the questionnaire. The content validity index per item ranged from 0.83 to 0.99, with an overall content validity score of 0.93 [25].

Patients were requested to rate their comfort level in one of three categories: “bad”, “good”, or “excellent”. The absence of pain or discomfort was classified as “excellent”; mild discomfort as “good”, and moderate to severe discomfort was considered as “bad”.

Socket discharge was categorized into three levels: mild, moderate, and severe, based on the amount of discharge observed. Lacrimation was assessed with a simple “yes” or “no” response. Similarly, lubrication frequency was categorized as “frequent” or “not frequent”. An increased need for lubrication indicated dryness and discomfort. Patients requiring lubrication 2–3 times daily were classified as “not frequent”, while those needing lubrication more than three times daily were classified as “frequent”.

### Microbiological analysis

Swabs for microbiological evaluation were collected from the conjunctiva of the defective socket in both groups before the insertion of the prosthesis and again three months later, following the removal of the artificial eye using a suction cup. Prior to sample collection, patients were instructed not to use antibiotics, either topically or systemically, for at least five days before the sample collection and to retain from washing the area with any antiseptic solution other than saline.

Sterile rayon swabs (Rayswab, Difco) were soaked in 9% sodium chloride from the inferior fornix of the anophthalmic cavity. Each swab was then cut off and placed in a tube (Becton, Dickinson & Company, Franklin Lakes, NJ, USA) containing 4 mL of sterile Brain Heart Infusion Broth (BHI). The tube was sealed, and cultures were promptly transported to the laboratory.

Isolates were identified through microscopy, culture methods, and biochemical tests. The samples underwent the procedures of conventional microbiology diagnostic method [26]. In the microbiology laboratory gram stained smear and culturing on different microbiological medium was done (blood agar. MacConkey’s agar and sabaroud dextrose agar) to be able to isolate the gram positive or gram negative bacteria and fungi from each specimen.

After 48 h incubation the colonies appeared will be subjected to biochemical tests to be identified. For isolates grew on blood agar only gram film from colonies was done and gram positive cocci was further identified using catalase test, bile esculine test, side and tube coagulase test.

For isolates grew on blood agar and macck agar gram stained film was done to confirm that it is a gram negative bacilli and the biochemical tests were done (TSI triple sugar iron agar, urease test, citrate test, and MIO-test) for non-lactose fermenting colonies oxidase test was added.

Gram stain film from colonies appeared on SDA was done. After the prescribed incubation period, the identification of the isolate was carried out through Gram staining, microscopy, and biochemical reactions.

### Blinding

A double-blinding strategy was employed in this trial. Both the patients and evaluators were blinded. Evaluators were blinded because pre-prosthetic and post-prosthetic swabs were taken when the prosthesis was not present in the socket. Because the construction methods employed for the conventional and digital ocular prostheses are totally different, it was impossible to blind the operators.

### Statistical analysis

Descriptive statistics were calculated as frequencies and percentages. Comparisons of microbial growth between the two study groups were conducted using the Fisher exact test. The McNemar test was employed for comparisons of microbial growth before and after the intervention within each group. Intention-to-treat analysis was used for analyzing all subjects in the current study. Significance was set at *p*-value < 0.05. Data analysis was performed using IBM SPSS for Windows (Version 26.0).

## Results

Based on the inclusion and exclusion criteria, 22 patients were included in this clinical trial, 11 in each group. After a 3-month follow-up, the data were collected and applied for the statistical analysis. All patients completed the study without dropping out of any patient after randomization.

### Questionnaire

**Fig. 4:**
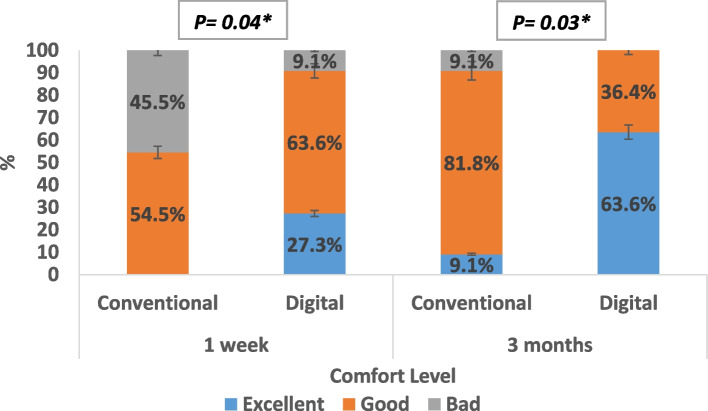
Comfort level

A statistically significant difference was recoded regarding the level of comfort between the two groups, both after one week of prosthesis insertion (*p* = 0.04) and after three months of prosthesis use (*p* = 0.03). Patients with digital ocular prostheses exhibited a higher level of comfort (Fig. 4).

**Fig. 5:**
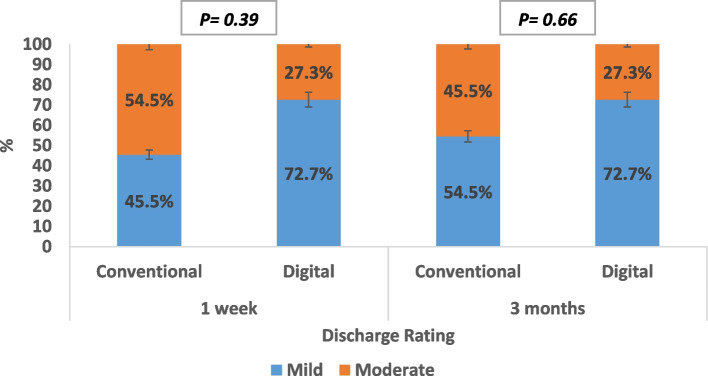
Discharge rating

As for discharge rating, there was no statistically significant difference observed between both groups, either after one week of prosthesis insertion (*p* = 0.39) or after three months of prosthesis use (*p* = 0.66). While both groups experienced a decrease in discharge percentage after three months of prosthesis use, none of the patients in either group reported severe discharge at any point during the study period (Fig. 5).

**Fig. 6:**
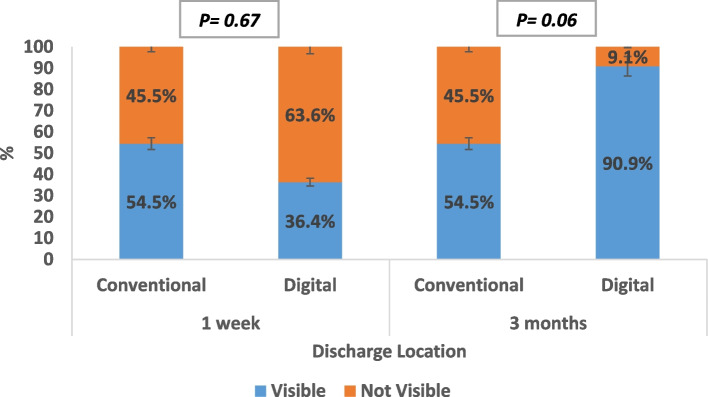
Discharge location

**Fig. 7:**
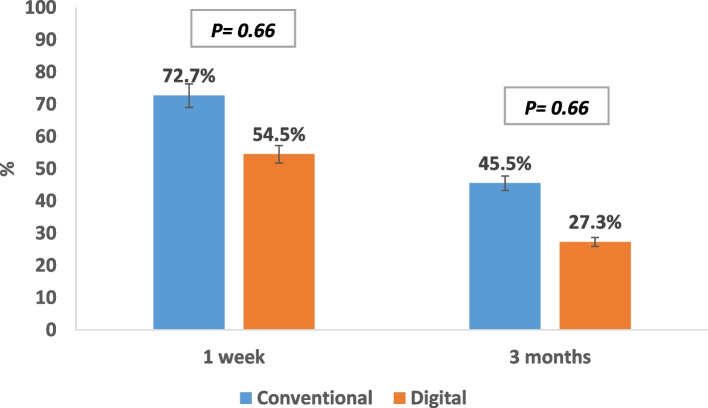
Lacrimation

**Fig. 8:**
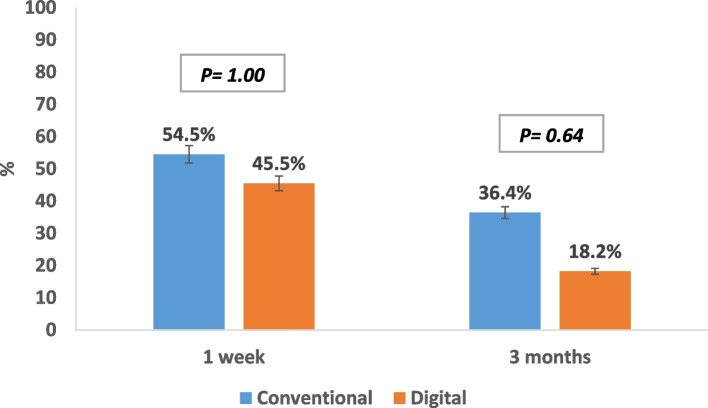
Lubrication frequency

Regarding discharge location, no statistically significant difference was recorded (Fig. 6).

Concerning lacrimation, no statistically significant difference was noted, although most patients in both groups showed improvement in lacrimation after three months of prosthesis use (Fig. 7).

In terms of lubrication frequency, there was no statistically significant difference between both groups. However, the needed lubrication frequency decreased after three months of prosthesis use in both groups (Fig. 8).

### Microbiological analysis

**Fig. 9:**
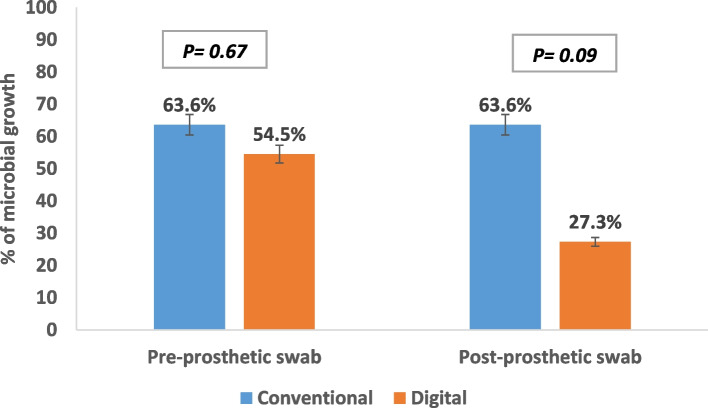
Microbial growth in the two study groups

In Group II, a noticeable reduction in microbial growth was observed after three months of prosthesis utilization. Initially, 54.62% (6 out of 11 patients) of pre-prosthetic swabs indicated microbial growth, which decreased to 27.3% (3 out of 11) after three months of prosthesis use. Conversely, Group I exhibited an unaltered percentage of microbial growth, remaining at 63.6% both before and after three months of prosthesis use (Fig. 9). Despite Group II demonstrating superior results in terms of reduced microbial growth post-prosthesis use, no statistically significant differences were observed in microbial growth either within each group before and after (*p* = 1.00) or between the conventional and digital groups after prosthesis use (*p* = 1.00) (Table 2).

**Table 2:** Comparison of microbial growth before and after the intervention between the two study groups

			Conventional	Digital	*P* value 1
			N (%)		
**Gram + ve cocci**	**Enterococci**	**Before**	1 (9.1%)	0 (0%)	1.00
		**After**	0 (0%)	0 (0%)	1.00
		***P*** **value 2**	1.00	1.00	
	**Coagulase -ve staphylococci**	**Before**	1 (9.1%)	2 (18.2%)	1.00
		**After**	1 (9.1%)	0 (0%)	1.00
		***P*** **value 2**	1.00	0.50	
	**Staphylococcus aureus**	**Before**	1 (9.1%)	3 (27.3%)	0.59
		**After**	2 (18.2%)	1 (9.1%)	1.00
		***P*** **value 2**	1.00	0.50	
	**MRSE**	**Before**	0 (0%)	0 (0%)	1.00
		**After**	1 (9.1%)	0 (0%)	1.00
		***P*** **value 2**	1.00	1.00	
**Gram + ve rods**	**Coryneform bacteria**	**Before**	1 (9.1%)	1 (9.1%)	1.00
		**After**	1 (9.1%)	0 (0%)	1.00
		***P*** **value 2**	1.00	1.00	
**Gram -ve rods**	**Pseudomonas spp.**	**Before**	0 (0%)	0 (0%)	1.00
		**After**	2 (18.2%)	2 (18.2%)	1.00
		***P*** **value 2**	0.50	0.50	
	**Klebsiella spp.**	**Before**	1 (9.1%)	0 (0%)	1.00
		**After**	0 (0%)	0 (0%)	1.00
		***P*** **value 2**	1.00	1.00	
**Fungal infection**		**Before**	2 (18.2%)	0 (0%)	0.48
		**After**	2 (18.2%)	0 (0%)	0.48
		***P*** **value 2**	1.00	1.00	

*P*-value 1: Comparisons of values between conventional and digital groups

*P*-value 2: Comparisons between values before and after within each group

Regarding bacterial analysis, gram-positive cocci species identified included Staphylococcus aureus, coagulase-negative staphylococci, Enterococci, and Methicillin-Resistant Staphylococcus epidermidis (MRSE). Gram-positive rods, specifically Coryneform bacteria, were also identified, along with gram-negative rods (Pseudomonas spp. and Klebsiella spp.).

In group I (CG), 27.3% of patients (3 out of 11) exhibited gram-positive cocci in pre-prosthetic swabs, which increased to 36.4% after three months of prosthesis use. In contrast, in Group II, 45.5% of patients initially had gram-positive cocci in pre-prosthetic swabs, but this percentage decreased to 9.1% (1 out of 11) after three months of prosthesis use. Notably, Coryneform bacteria were the only gram-positive rods detected. Two patients were found to have Coryneform bacteria; one patient in Group I had Coryneform bacteria in the pre-prosthetic swab, which persisted at the three-month evaluation swab, while another patient (9.1%) in the same group had Coryneform bacteria in the pre-prosthetic swab but not after three months of prosthesis use.

Klebsiella spp. was detected in only one patient in Group I (CG) (9.1%) and was not found in the three-month post-prosthetic swab. It was not detected in Group II (DG) either before or after prosthesis use.

Pseudomonas spp. was not detected in either group in the pre-prosthetic swab. However, it was detected in 18.2% (2 out of 11) of patients in Group I and also in 18.2% in Group II in the three-month post-prosthetic swab.

Candida was isolated in 18.2% of patients (2 out of 11) in Group I and persisted at three-month evaluation swabs. In Group II, no fungi were isolated either before or after prosthesis use.

Figure 9 Shows frequencies and percentages were calculated for all variables. Comparisons between the two study groups were conducted using the chi-square test with Monte Carlo corrected p-values and Fisher’s exact tests.

## Discussion

While various factors can influence the comfort level of ocular prosthesis patients, the disturbance of ocular flora may be the most crucial factor affecting their ocular comfort. Previous studies have examined changes in microbial flora within anophthalmic sockets. However, the majority of these studies focused on comparing the flora of the anophthalmic socket with that of a healthy eye [3, 4, 27, 28].

All of these studies have reported a higher level of microorganism formation in the anophthalmic cavity and ocular prosthesis compared to the contralateral eye. This increased presence was observed for total bacteria, S. aureus, S. epidermidis, and Candida albicans. Staphylococcus species are significant pathogens associated with prosthetic infections. Furthermore, the anophthalmic cavity provides an ideal environment for the proliferation of undesirable fungi, especially Candida albicans yeast [2, 29].

Some studies have examined the effects of various cleansing methods, the frequency of ocular prosthesis manipulation, the age of the ocular prosthesis, the frequency of polishing, and the administration of antibiotic eye drops on the flora of the socket and ocular prosthesis biofilm [4, 17, 30–33]. All these factors contribute to disturbances in the socket flora.

Since all patients in both groups received their prostheses for the first time and were assessed three months later to eliminate the age of the prosthesis as a factor, all patients in our study underwent the same cleaning procedures for their ocular prostheses. This cleaning regimen included washing them with water and soap once a week. Furthermore, none of the prostheses were polished within the first three months of use, and all patients were instructed not to use antibiotic eye drops.

A number of earlier studies [34–37] have examined the impact of the materials used in ocular prostheses. Most of these studies have focused on polymethyl methacrylate, or PMMA, which is believed to be the most commonly used material in ocular prosthesis fabrication. They have investigated its effect on issues such as discharge, irritation, and inflammation of the anophthalmic socket. These problems are primarily caused by disruptions to the microbial flora of the socket and the formation of biofilm on the ocular prosthesis. However, there is limited information available on whether ocular prostheses manufactured from 3D-printed resins are susceptible to bacterial adhesion and biofilm formation.

The results of the current study showed a lower percentage of microbial growth in Group II (digitally designed 3D-printed) ocular prostheses, with (72.7) % of sockets showing no growth after three months of prosthesis use. This is in contrast to a higher percentage of microbial growth observed in Group I patients (conventional heat cured PMMA). Although Group II shows positive results, there is no statistically significant difference between the two groups and the null hypothesis was accepted. We observed a positive correlation between the material and design of the prosthesis and changes in microbial flora. This could be explained by the effect of surface roughness, which is considered one of the key factors influencing the adhesion and colonization of microorganisms on biomaterials. Depressions, micro-cracks and porosities in roughened surfaces provide more favorable colonization sites [14, 38–40]. Most studies have revealed a strong positive correlation between surface roughness and the number of viable bacteria. These results align with findings reported by other authors [14, 37, 38], such as Gad et al. [41], who found significant differences in all tested properties between heat-polymerized and 3D-printed denture base materials,3D-printed resin showed superior surface roughness. The surface roughness values of non-thermally cycled 3D-printed resin fell within the maximum clinically acceptable value of 0.2 μm [41]. Wuersching et al. [42] investigated the initial bacterial adhesion on 3D-printed splint materials in relation to their surface properties. Specimens included two conventional powder/liquid PMMA materials, and results showed that the 3D-printed splints exhibited overall favorable results regarding surface roughness and bacterial adhesion. Regarding Candida, Murat et al [43] compared the amount of adherent Candida albicans to different CAD/CAM poly (methyl methacrylate) (PMMA)-based polymers and conventional heat-polymerized PMMA. Their results showed that CAD/CAM PMMA-based polymers had less surface roughness and less adhesion of Candida albicans. Mazurek et al. [44] evaluated biofilm formation on 3D-printed temporary restorations and assessed the post-processing impact on microbial adhesion. They found that roughness itself was not the main factor affecting microbial adhesion to 3D-printed resin. Instead, post-processing mechanisms played a significant role, with glazed specimens showing the best results in reducing bacterial adhesion, followed by polished and raw specimens, which exhibited a higher percentage of bacterial adhesion.

The findings of the current study, along with those of previous studies, clarify that Group II patients have significantly higher levels of comfort than Group I patients. Group II patients also experience less discharge, although both groups’ discharge rates started to decline after three months of use.

In contrast, some studies have reported opposing results. Teixeira et al. [45], evaluated the adhesion of multispecies biofilm, surface characteristics, flexural strength, and elastic modulus of heat-cured resin incorporated with AgVO3 compared to conventional heat-cured and printed resins. Printed resin for denture bases showed higher irregularities, a metabolically active biofilm, lower flexural strength, and elastic modulus than heat-cured resin. Additionally, in a study by Meirowitz et al. [46], results showed that the microbial cell counts adhered to the 3D-printed discs were significantly higher compared to the heat-cured samples, whereas the milled samples showed significantly lower counts. In a study conducted by Schubert et al. [47], results showed that three-dimensional printing and pressing were associated with significantly higher C. albicans adhesion than thermoforming. For S. mutans adhesion, there were no significant differences between the resins or between the manufacturing methods. The results also indicated a slightly positive but statistically insignificant correlation between surface roughness and microbial adhesion.

Another important factor in reducing microbial growth is the fit and design of the ocular prosthesis, which plays a crucial role in eliminating dead space between the prosthesis fitting surface and the anophthalmic socket. This eliminates the accumulation of secretions and creates an environment less favorable for microbial growth. The lower percentages of microbial growth in Group II can be explained by the precision of 3D-printing and software design technology, as reported by other authors who found that 3D-printed restorations exhibited higher accuracy and better fit. Aldahian et al. [48], investigated the influence of fabrication techniques on the marginal fit, adaptation, surface roughness, and wear of interim restorations. Their findings indicated that 3D-printing showed superior efficacy compared to CAD-CAM and conventional techniques in terms of marginal fit, adaptation, and surface wear. Al Deeb et al. [49] assessed the marginal fit, internal adaptation, and compressive strength of SLA Provisionals (SLA) compared to CAD-CAM and conventional (CONV) interim fixed partial dentures (FPDs). CAD-CAM and SLA Provisionals showed comparable marginal fit and internal adaptation, which were significantly higher than the conventional group, which exhibited the lowest values. The misfit of conventional heat-cured restorations may be attributed to the manual preparation of the restoration, trimming of excess resin, dimensional contraction during the polymerization reaction, and exothermic reactions causing additional contraction when the restoration cools [50].

The insignificant difference between the two groups may be attributed to the hydrophobicity of both materials. Several studies have demonstrated the importance of the material’s hydrophobic effect on initial adhesion, showing a linear correlation between an increase in hydrophobicity and the number of attached cells [51, 52]. Some studies have also shown that the number of viable cells within the biofilm can differ despite similar surface roughness. This suggests other parameters, such as surface free energy, stiffness, charge density, and polarity [47–53] may affect microbial adhesion, explaining the insignificant difference between the two groups regarding lacrimation and lubrication frequency, which are related to the hydrophobicity and wettability of the material. However, both groups showed improved lacrimation and frequency of lubrication after use, as all patients required a considerable period of adaptation to their new prosthesis.

Pseudomonas spp. were recorded in the post-prosthetic swabs of four patients, two from Group I (CG) and two from Group II (DG). However, their pre-prosthetic swabs were free from Pseudomonas spp. This can be primarily attributed to the socio-cultural environment of the patients, which is directly related to their hygiene habits. This airborne bacterium is mainly introduced to the socket during manipulation of the prosthesis with inadequate hygienic habits of the patients. These habits are not only related to their socio-economic status but may also be influenced by their occupation [54].

P. aeruginosa can also effectively colonize a variety of surfaces, including most medical materials, and is a well-known biofilm former. This biofilm acts as a scaffold for adhesion to the prosthesis surface and protects it from the surrounding environment. Biofilms of P. aeruginosa are composed of polysaccharides and extracellular DNA, which play critical roles in protecting the bacterial communities from exogenous stresses caused by antimicrobial agents [55].

In Group I, two patients had positive pre-prosthetic swabs. One had Klebsiella spp., and the other had Enterococci. However, both had negative post-prosthetic swabs. This can be explained by considering both patients as treated individuals who used prophylactic antibiotic eye drops prescribed during the first week of prosthesis use.

The limitation of this RCT was the short follow-up period restricted to 3 months. More RCTs are needed with longer follow-up periods to allow for evaluation of long term effect of the material and design of 3D-printed ocular prosthesis on the health of the anophthalmic socket.

## Conclusions

From the current study we can conclude the following:

- Digitally designed 3D-printed customized ocular prosthesis are considered a successful treatment modality for treating anophthalmic patients.
- Patients with 3D-printed ocular prostheses had a lower percentage of microbial growth in their sockets compared to the conventional PMMA wearers, although the difference was not staistically significant. However, patients with digital prostheses reported significantly higher comfort levels than conventional prosthesis wearers.
- Regardless of the construction method, all patients require an adaptation period with their prostheses. Over time, they all show improvements in terms of comfort, discharge, lacrimation, and the need for lubrication.

## Data Availability

The datasets used and analyzed during the current RCT are available from the corresponding author upon reasonable request.

## Abbreviations

3D: Three‑Dimensional
PMMA: Poly-Methyl Methacrylate
CAD: Computer Aided Design
CAM: Computer Aided Manufacture
SDA: Sabouraud’s Dextrose Agar
BHI: Brain Heart Infusion Broth
CT: Computerized Tomography
DICOM: Digital Imaging and Communications in Medicine
MIMICS: Materializes Interactive Medical Image Control System
SLA: Stereolithography
STL: Standard Tessellation Language
MRSE: Methicillin-Resistant Staphylococcus Epidermidis
